# A plug-in electrophoresis microchip with PCB electrodes for contactless conductivity detection

**DOI:** 10.1098/rsos.171687

**Published:** 2018-05-09

**Authors:** Mingpeng Yang, Zhe Huang, Hui You

**Affiliations:** 1 Institute of Intelligent Machines, Chinese Academy of Sciences, Hefei 230031, Anhui, People's Republic of China; 2 University of Science and Technology of China, USTC, Hefei 230026, Anhui, People's Republic of China

**Keywords:** electrophoresis, microchip, plug-in structure

## Abstract

A plug-in electrophoresis microchip for large-scale use aimed at improving maintainability with low fabrication and maintenance costs is proposed in this paper. The plug-in microchip improves the maintainability of a device because the damaged microchannel layer can be changed without needing to cut off the circuit wires in the detection component. Obviously, the plug-in structure reduces waste compared with earlier microchips; at present the whole microchip has to be discarded, including the electrode layer and the microchannel layer. The fabrication cost was reduced as far as possible by adopting a steel template and printed circuit board electrodes that avoided the complex photolithography, metal deposition and sputtering processes. The detection performance of our microchip was assessed by electrophoresis experiments. The results showed an acceptable gradient and stable detection performance. The effect of the installation shift between the microchannel layer and the electrode layer brought about by the plug-in structure was also evaluated. The results indicated that, as long as the shift was controlled within a reasonable scope, its effect on the detection performance was acceptable. The plug-in microchip described in this paper represents a new train of thought for the large-scale use and design of portable instruments with electrophoresis microchips in the future.

## 1. Introduction

Microchip electrophoresis (ME) is emerging as a highly promising method for rapid analysis with a minimum amount of analytes [1–4]. Compared with other ion detection methods such as laser-induced breakdown spectrometry [5] and inductively coupled plasma-mass spectrometry [6], ME does not require expensive equipment and complex mechanisms and is expected to achieve real portability. When a voltage is applied at the two ends of a microchannel filled with solution, the ions in the solution are moved and separated because of the effects of electro-osmotic flow and electrophoresis [7]. According to the different arrival times of various ions and the signal intensity detected, the species and concentrations of the ions can be recognized. ME has been used in many fields, such as environmental detection, soil nutrient testing, medical diagnosis and biotechnology [8–12].

Creating such an electrophoresis microchip has always been a hot research topic [13–15]. The electrophoresis microchip usually has two parts, a microchannel layer and an electrode layer [16,17]. Usually, organic materials such as polydimethylsiloxane (PDMS), polymethylmethacrylate (PMMA) and polycarbonate are used preferentially to make the microchannel layer because of advantages such as ease of accessibility, ease of manufacturing and inexpensive cost. Standard photolithography and/or the subsequent hot-embossing process and bonding process are used to form the microchannel layer [18,19]. The fabrication processes are time consuming and expensive. Also, the convex template made of SU8 [16] or PDMS [20] that used in the hot-embossing process has a short life because of its weak material strength and bonding strength.

The complex manufacturing process of the electrode layer is another unavoidable problem, and is even more involved than that for the microchannel layer. Usually, typical photolithography and metal deposition or sputtering processes are needed to finish the manufacturing process and precious metals such as platinum or gold are consumed simultaneously [19,21,22]. Because of the complex manufacturing process required for the electrode layer, a lot of work was carried out by many researchers. Zhao *et al*. [23] proposed using detection electrodes made of indium tin oxide (ITO)-coated glass to avoid the intricate electrode-manufacturing processes. The electrodes were fabricated by screen printing and etching processes using an ITO-coated glass wafer. This method reduced the electrode fabrication cost and simplified the electrode manufacturing process, but the method was still complicated because of the requirement for photolithography and etching processes. Thredgold *et al*. [24] presented ‘injected' metal electrodes for their electrophoresis microchip. Two electrode channels were fabricated at the microchannel layer together with the injection channel and separation channel. Then molten gallium was injected into the electrode channels at a temperature below the PMMA transition point but higher than the melting point of gallium. The ‘injection' electrodes simplified the electrode fabrication process significantly, but photolithography was still needed.

Another fatal flaw of the electrophoresis microchip is its short life and poor maintainability. Blockages or leakages frequently occur in the microchannel layer when the microchip is in use. The short life of paper-based chips is especially striking. Electrophoresis microchips are used only once in a lot of laboratories, and their short life and high fabrication cost are the most important reasons why their use is not widespread. Usually, the microchannel layer and the electrode layer are bonded together by a hot-bonding or plasma-bonding process [19,25]. This means that, when the microchannel layer stops working normally, the whole microchip, including the microchannel layer and the electrode layer, has to be discarded. It is a painful waste. A lot of researchers have tried to integrate an electrophoresis microchip into a mobile detection instrument that can be used outside the laboratory to realize *in situ* detection [9,26]. However, when the microchip breaks down, replacing it is a challenge for untrained users because they need to cut off all the circuit wires on the detection component and reconnect them again.

On the basis of previous work, we proposed an electrophoresis microchip for large-scale use aimed at improving maintainability with low fabrication and maintenance costs. A plug-in structure with the microchannel layer fixed in a clamp is described in this paper. The clamp was fixed on the printed circuit board (PCB) plate and the microchannel layer was independent of the electrode layer. When the microchannel layer leaks or is blocked, it can be just pulled out and replaced with a new one. This greatly improves the maintainability and lowers the maintenance cost. Also, various approaches were employed to lower the fabrication cost and reduce the manufacturing time. The detection performance and the effect of the installation shift brought about by the plug-in structure are also discussed in this paper.

## 2. Material and methods

### 2.1. Materials, reagents and samples

Histidine (His), 2-(*N*-morpholino) ethanesulfonic acid (MES) and 18-crown-6 were purchased from Hefei Baierdi Chemical Technology Co. Ltd, China. Potassium chloride, sodium chloride and lithium chloride were purchased from Sinopharm Chemical Reagent Co. Ltd, China. All reagents were of analytical grade, and deionized water was used throughout.

Two high-voltage modules (DW-P102-1C32 and DWP502-1C0F; Dongwen Cop., China; http://www.tjindw.com) were used to supply the injection and separation voltages for the electrophoresis experiments. A CNC milling machine (SXX05; Sharpe CNC Co. Ltd, China; http://www.sharpecnc.com) was employed to fabricate the steel template with a convex microchannel pattern. An ultrasonic cleaner and a Harrick Plasma Cleaner (PDC-002) were used to clean and active the surfaces of the PMMA plates. A self-made hot-embossing device and a heating furnace were introduced to copy microchannels from the steel template and seal the microchannel layer.

### 2.2. Apparatus

**Figure 1.**
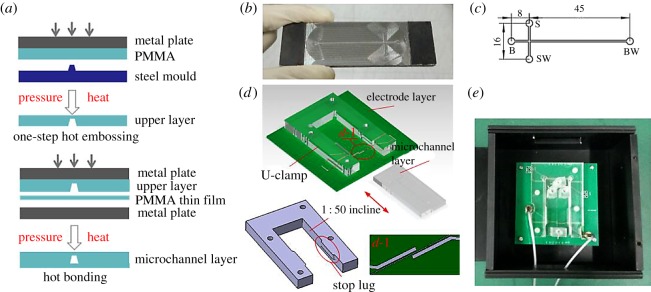
Fabrication and installation of the electrophoresis microchip. (*a*) The fabrication processes including hot embossing and hot bonding. (*b*) The real metal template produced with a CNC milling machine. (*c*) The size of the microchannels. S, sample reservoir; SW, sample waste reservoir; B, buffer reservoir; BW, buffer waste reservoir. (*d*) Right upper: Insertion and pull out of the microchannel layer. Left bottom: The U clamp used to fix the microchannel layer. The detailed structure of the electrodes is described in (*d*-1). (*e*) A plug-in microchip including the microchannel layer and the electrode layer.

A CNC graphing tool (Mastercam 9.1) was employed to draw the microchannel pattern and generate the CNC programs for the manufacture of the steel template. Because the pattern of the steel template was very elaborate, a milling cutter with a diameter of 0.5 mm and a high rotation speed of 15 000 r.p.m. were used. The hot-embossing and the hot-bonding processes are illustrated in figure 1*a*. A PMMA plate with thickness 5 mm was put on the steel template at a pressure of 0.6 MPa and temperature of 103°C, a little lower than the glass transition temperature of PMMA, for 40 min. Then it was naturally cooled to room temperature and the pattern of the steel template was copied to the PMMA plate. An ultrasonic cleaner was employed to rinse the PMMA plate and another 200 μm thickness PMMA thin film with deionized water for 15 min. The cleaning process was repeated three times and the deionized water was changed each time. Before bonding the two PMMA plates, they were treated with a plasma cleaner (1 min, high power) to improve the surface activity. Finally, the two PMMA plates were subjected to a pressure of 0.6 MPa and temperature 90°C for 30 min to finish the hot-bonding process. A sealed microchannel layer was achieved after naturally cooling to room temperature. The size of the microchannel layer was 30 mm × 70 mm and the effective separation length was approximately 45 mm, as shown in figure 1*c*. The designed width and depth of the microchannels were 100 µm and 100 µm, respectively. The length of the injection channel was 8 mm. The diameter of the reservoirs was 2 mm.

**Figure 2.**
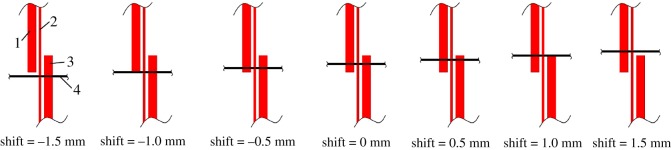
Schematic diagram of the installation shift between the microchannel layer and the electrode layer. Seven shifts were defined, ranging from −1.5 to 1.5 mm. 1, signal-sending electrode; 2, grounding electrode; 3, signal-receiving electrode and 4, microchannel.

The PCB plate with detection electrodes was manufactured by J&C Co. Ltd, China. The PCB manufacturing process is a fairly mature technology and has been widely used for decades. The manufacturing cost of common PCB plates is cheap because of their mass production. The detection electrodes were patterned on the PCB plate. We chose a three-electrode structure containing a signal-sending electrode, a signal-receiving electrode and a grounding electrode (see figures 1 and 2). The three-electrode structure can provide a better detection performance than the two-electrode structure [24,27,28]. The height of the three electrodes was 35 µm. The widths of the signal-sending electrode and the signal-receiving electrode were both 1 mm. The width of the grounding electrode was 0.2 mm. The gaps between the three electrodes were both 0.3 mm.

A U clamp was designed and manufactured to secure the microchannel layer. As shown in figure 1*d*, in order to fix the microchannel layer, the inner sides of the clamp were designed as a 1 : 50 incline. Two stop lugs were also employed to limit the movement of the microchannel layer in the vertical direction. The clamp was assembled on the PCB plate by four plastic bolts firstly. And then we just need to insert the microchannel layer or pull it out of the clamp to install or remove the microchannel layer.

### 2.3. Electrophoresis procedure

Before electrophoresis, the microchannels were washed with deionized water and running buffer (20 mM MES/His–0.7 mM 18-crown-6) for 20 min, respectively. All ion samples, including potassium chloride, sodium chloride and lithium chloride, were dissolved in running buffer. Sample injection was performed electrokinetically using the normal cross-injection method [29]. A voltage of 500 V was applied between reservoir S and reservoir SW for 10 s, as shown in figure 1*c*. Sample separation was performed by applying a voltage of 1000 V between reservoirs B and BW. The voltage and frequency of the excitation signal were 5 V_pp_ and 800 kHz, respectively.

### 2.4. Electrophoresis experiment for the installation shift

The plug-in structure brings excellent maintainability; however, it causes another problem with the installation shift between the separation channel and the electrodes because the plug-in structure cannot guarantee precision assembly of the microchannel layer and the electrode layer. To evaluate the effect of installation shift on the detection performance, a set of comparison experiments were conducted.

As shown in figure 2, a series of installation shifts were defined from −1.5 mm to 1.5 mm, and electrophoresis detection was implemented for each installation shift, respectively. A total of 0.5 mM mixed solution of K^+^, Na^+^, Li^+^ was chosen to assess the effect of the installation shift on the test performance. The experimental method and parameters were as described in §3.3.

## 3. Results and discussion

### 3.1. Quality of the microchannels

**Figure 3.**
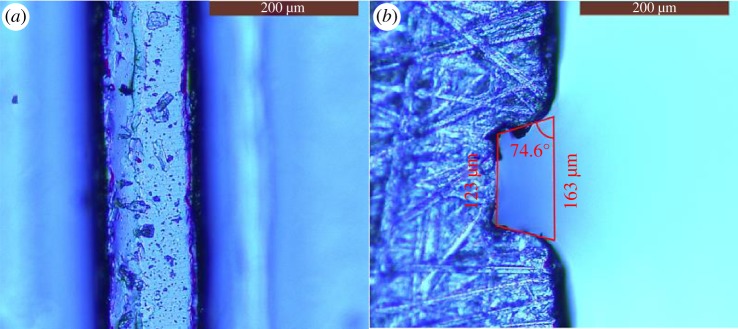
Micrographs of the microchannel layer copied from the metal template. (*a*) Top view image of the microchannel. (*b*) Section view image of the microchannel.

To evaluate the quality of the microchannels made from the metal template, the upper layer of the PMMA microchannel layer was observed by microscope (Leica DMI3000 M). Several bumps and hollows with a size of several micrometres were observed, as shown in figure 3*a*. They were copied from the bumps and hollows of the metal template and were inevitable because machining cannot guarantee the excellent surface smoothness that is possible with photolithography. The layer was cut off and smoothed with a piece of fine sandpaper, then the cross section of the microchannel was obtained. It can be seen that the cross section is an approximate trapezoid, as shown in figure 3*b*. The topline and the baseline are 123 µm and 163 µm, respectively. The angle of the lower corner is approximately 74.6°. The height of the trapezoid is approximately 70 µm. The designed size of the microchannel section was 100 µm × 100 µm and the discrepancy was caused by the hot-embossing and demoulding processes.

### 3.2. Detection performance

**Figure 4.**
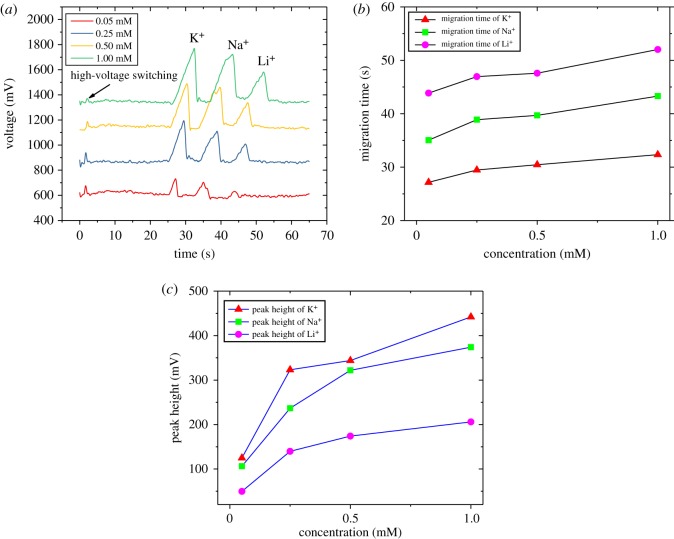
Electrophoresis test results with different concentrations of K^+^, Na^+^ and Li^+^. Running buffer: 20 mM MES/His–0.7 mM 18-crown-6; injection voltage 500 V for 10 s; separation voltage 1000 V; detection at 800 kHz and 5 V_pp_. (*a*) Electrophoretogram for a mixture containing K^+^, Na^+^ and Li^+^ at concentrations of 0.05, 0.25, 0.5 and 1 mM. (*b*) Effect of the concentration on the migration time. (*c*) Effect of the concentration on the peak height.

To evaluate the detection performance of our microchip, a series of experiments were performed. A set of experiments for the gradient concentration test were conducted. Several equimolar mixtures of K^+^, Na^+^ and Li^+^ dissolved in the running buffer (20 mM MES/His–0.7 mM 18-crown-6) were tested by employing the plug-in microchip. In the detection electrophoresis diagram shown in figure 4*a*, 0.05 mM, 0.25 mM, 0.50 mM and 1.0 mM mixed solutions were absolutely separated, respectively, and defined in the order of K^+^, Na^+^ and Li^+^. The peak height and the migration time when the three ions arrived at the detection position were extracted from the experimental data. From figure 4*b*,*c*, several features of the ion migration can be seen. At first, the migration speeds of the three ions are diverse and in the order of K^+^, Na^+^ and Li^+^, which is consistent with results in the literature [24]. For the same ion at different concentrations, the migration speed slightly decreases with increasing concentration. The peak height of the three ions at the same concentration is in the order of K^+^, Na^+^ and Li^+^. The peak height of the same ion at different concentrations increases with the increase in the concentration and shows an acceptable linear relationship between concentration and peak height at concentrations ranging from 0.25 to 1 mM.

**Figure 5.**
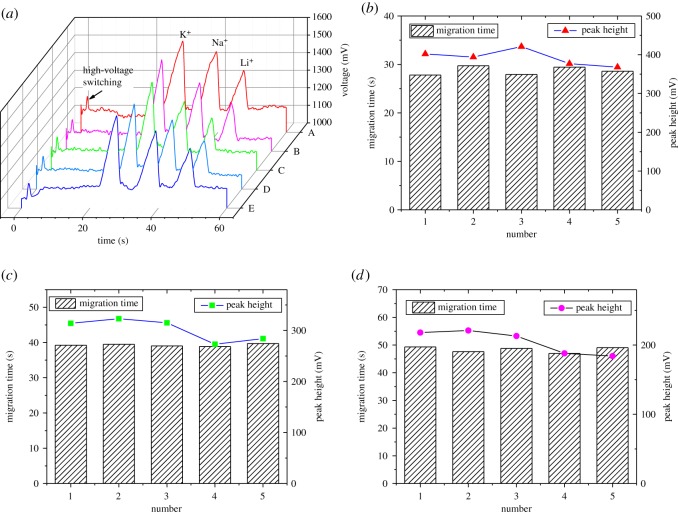
Repeatability of K^+^, Na^+^, Li^+^ (0.5 mM each) in five tests. Running buffer: 20 mM MES/His–0.7 mM 18-crown-6; injection voltage 500 V for 10 s; separation voltage 1000 V; detection at 800 kHz and 5 V_pp_. (*a*) Electrophoretogram of K^+^, Na^+^, Li^+^ in five tests. (*b*–*d*) Migration time and peak height of K^+^ (triangles), Na^+^ (squares), Li^+^ (circles) in five tests, respectively.

To assess the test stability of the plug-in microchip, several detection tests of the three ions K^+^, Na^+^, Li^+^ at a concentration of 0.5 mM were carried out. The results are shown in figure 5*a*. The peak height and migration time of each ion were extracted, which indicated a good consistency, as shown in figure 5*b–d*. The average and the repeatability of the migration time are listed in table 1. The average and repeatability of the peak height are also listed. Compared with [30,31], the repeatability of the migration time and the peak height are at the same level. In addition, the limits of detection (LODs) for K^+^, Na^+^ and Li^+^ were obtained through experimental detection. In order to obtain more persuasive results of the LODs for the three ions, several experiments were conducted. The LOD for K^+^ is taken as an example for the explanation of the experiments. The detection experiments of K^+^ at concentrations of 0.25 mM, 0.20 mM and 0.15 mM were implemented, respectively. K^+^ at concentrations higher than 0.20 mM could be detected; however, K^+^ at a concentration of 0.15 mM could not be recognized clearly in the electrophoretogram. We also considered 0.20 mM as the LOD for K^+^. However, the real LOD for K^+^ was between 0.15 and 0.20 mM, and we chose the higher one for a conservative evaluation. Similar detection experiments were conducted for Na^+^ and Li^+^. In summary, the LODs for K^+^, Na^+^ and Li^+^ were 20 µM, 25 µM and 40 µM, respectively. Compared with other microchips [30,31], the LODs of the plug-in microchip were several times higher because of the thick insulating film used to seal the microchannel layer, the rough inner face of the microchannels and the assembled structure. Also some experimental parameters including buffer concentration and structure parameters were not systematically optimized.

**Table 1.** Main analytical parameters for the separation of K^+^, Na^+^, Li^+^ in figure 5.

	K^+^	Na^+^	Li^+^
average migration time (s)	28.7	39.2	48.3
repeatability (RSD %) – migration time	3.0%	0.8%	2.1%
average peak height (mV)	392	301	204
repeatability (RSD %) – peak height	5.3%	7%	8.5%

### 3.3. Effects of the installation shift

**Figure 6.**
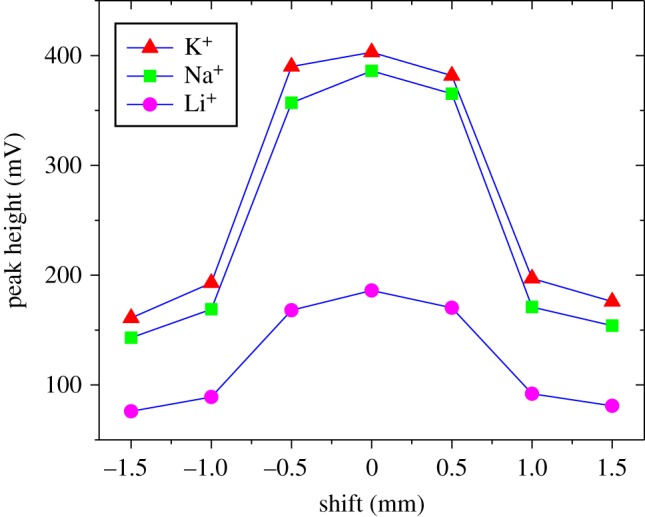
Electrophoresis test results for K^+^, Na^+^, Li^+^ with different installation shifts between the microchannel layer and the electrode layer. Experimental conditions were as described in the text. The concentrations of the ions were all 0.5 mM.

The effect of the installation shift brought about by the plug-in structure of our electrophoresis microchip, described in §3.4, was evaluated. The experimental results shown in figure 6 indicate that the installation shift between the microchannel layer and the electrode layer lowers the detection precision. To express the relationship between the installation shift and the detection performance, we define a parameter of shift distance that is the absolute distance from the zero shift position. With increasing shift distance, the detected signal intensity decreases obviously. However, the decrease in signal intensity caused by different shift distances was quite different. When the shift distance is greater than 1 mm, the signal intensity falls sharply and is below 50% of that at the zero shift position. However, when the shift distance is less than 0.5 mm, the signal intensity can be maintained within 95% of that at the zero shift position. So it can be concluded that, as long as the shift distance is controlled within 0.5 mm, the test results are acceptable. This is a meaningful result for the plug-in microchip, because it is the basis on which our chip can be effectively used. As long as the U-clamp and the microchannel layer can be fabricated with an accurate size, the assembling accuracy can be controlled within 0.5 mm easily. With the mechanical technology available at present, a machining tolerance of 0.25 mm for the small chip and clamp is easy to attain.

### 3.4. Service life

Six microchips were obtained using the hot-embossing and hot-bonding processes and numbered, respectively. The six microchips including the microchannel layer and the PCB plate were all employed in our electrophoresis experiments in succession. The experimental conditions including running buffer components, deionized water injection speed and time and buffer injection speed and time were consistent. The running buffer was 20 mM MES/His–0.7 mM 18-crown-6. The injection speed and time of deionized water and running buffer were all 0.1 ml min^−1^ and 20 min per time, respectively. The experimental conditions were the same as those described in §3.3. The service life of the six microchips was traced and is listed in table 2. The average service life of our microchips is approximately 35 times. The cost of one whole microchip is approximately $1.20 and the replacement cost of one microchannel layer is approximately $0.10. That is to say, the single-time using cost is approximately $0.003, which is a very low using cost.

**Table 2.** Recorded service life of six microchips.

	MC1	MC2	MC3	MC4	MC5	MC6
using times	29	38	43	25	36	39

## 4. Conclusion

The proposed method of our plug-in microchip improves maintainability greatly by using a plug-in structure to change the damaged microchannel layer without cutting the circuit wires on the detection component. This method lowers the maintenance cost because only the microchannel layer needs to be changed instead of the whole microchip, including the microchannel layer and the electrode layer. It also lowers the fabrication cost by adopting a steel template and PCB electrodes, avoiding the complex photolithography, metal deposition and sputtering processes. The detection performance of our microchip was also assessed by electrophoresis experiments. The results presented an acceptable gradient and a stable detection performance. The effect of the installation shift between the microchannel layer and the electrode layer brought about by the plug-in structure was also evaluated. The results indicated that, as long as the shift was controlled within a reasonable scope, its effect on the detection performance was acceptable. The LODs for K^+^, Na^+^, Li^+^ are relatively higher than previous results for other microchips; however, this does not reduce the application prospect and potential. This microchip can be used in many fields that do not need rigorous detection precision, such as detecting nutrient ions in soil [32] or monitoring the lithium ion concentration in the blood of patients with bipolar disorder who are treated with oral lithium [33]. The plug-in microchip described in this paper indicates a new train of thought for instrument design with electrophoresis microchips and large-scale use with relatively low cost in the future. Developing a detection instrument with a plug-in microchip will be the next step for our research team.

## Supplementary Material

Data of concentration grades used in figure 4

## Supplementary Material

Data of 5 repeative tests used in figure 5

## Supplementary Material

Data of test performance with different installation shifts

## Data accessibility

The datasets supporting this article have been uploaded as part of the electronic supplementary material.
